# Surgical Shock

**Published:** 1907-03

**Authors:** 


### SURGICAL SHOCK.

---

An enumeration of the causes of surgical shock is not necessary for the readers of this Journal, but it will perhaps be instructive to note some of the rarer forms of the condition, and then to consider, briefly, the general treatment and prophylaxis.

An interesting form of pure shock sometimes follows the sudden lowering of tension in a liver engorged by chronic damming back of the bile. An instructive case in point was that of a sixty year old woman deeply jaundiced for months on account of complete obstruction of the common duct by a large calculus. The patient was very much emaciated, and the stone—not recognized as such before operation—could be actually palpated through the unopened abdomen. Choledochotomy through a three-inch incision and extraction with suture of the opening in the duct was done in twenty minutes—a remarkably short time for this procedure. No hemorrhage ensued, but deep shock supervened at once with death in two hours. The writer has observed this form in other similar cases, but never again with a fatal ending.

Incision into one of the important body cavities often determines the occurrence of shock, its gravity being directly proportional to the extent of the opening, from the mere puncture of the walls of the cavity with no shock, to the enormous incision recommended by certain writers when discussing operations on the gall-bladder (Kehr, Riedel), in which the subsequent shock is one of the most dangerous features of the operation.

The occurrence of local shock is an important and interesting phenomenon. The writer has noticed this in operations about the kidney and in certain intra-abdominal work. He has seen so-called reflex suppression in a healthy kidney following operations upon its diseased fellow in spite of the almost total absence of the usual symptoms of general shock.

The parlytic intestinal distension immediately following the removal of very large abdominal tumors is a dangerous concomitant of local visceral shock, embarrassing the work of the heart and lungs and inviting intraperitoneal infection. This distension may even be great enough to cause obstruction of the intestinal canal through angulation.

We will now say a few words on the treatment and prophylaxis of shock. Agreement is general as to the necessity for combating the effects of diminished blood pressure by means of posture—inverting the patient—and by further increasing the amount of circulating arterial blood in the vital nerve centers by bandaging or even by intravenous saline infusion. In shock the large abdominal veins hold so much blood that the effect is practically the same as if it had been actually abstracted from the body. This may be partly overcome by firmly binding the abdomen and by centripetally bandaging the extremities. General pressure by the pneumatic suit of Crile accomplishes the result in an accurate and scientific manner. As to the value of intravenous infusion there is some divergence of opinion, but principally as to whether the infusion should be made early or late, after other measures have been vainly tried. It is evident that in shock associated with hemorrhage, perhaps even caused by it, normal conditions will be more speedily approximated by intravascular saline infusion. If, however, the loss of blood has been so great that the quality of the circulating fluid may not be expected to sustain life, then direct arteriovenous anastomosis, preferably with a brother or sister, may save the patient. Crile has tested this procedure, operating by Carrel's method with most striking results. In pure shock not associattd with great loss of blood the method is out of place. In the minor degrees it is well to avoid direct saline infusion, administering the fluid by stimulating saline enema or by the very valuable slow rectal infusion of Murphy, in which several quarts of saline solution are permitted to flow, drop by drop, into the rectum with the result that all is absorbed. Some years ago the intravenous method was pretty thoroughly tested by its employment in nearly every case of surgical shock. A number of postmortem examinations revealing edema of the kidneys made us more cureful, and now the method is reserved for the most desperate cases only, the others being treated through the rectum, or by hypodermoclysis. .

We now come to the discussion of the treatment by drugs, and here we have the widest divergence of opinion, many writers vigorously denouncing what others consider their most valuable remedies. Strychnin, digitalis, atropin and alcohol are alternately lauded and condemned to the great confusion of the student. To cite an example, strychnin, which has been recommended by numerous authors as invaluable, is pronounced by J. P. L. Mummery (Lancet, April 1, 1905) as most dangerous. He states that strychnin alone has been known to produce shock. The alkaloid which can be most depended upon is undoubtedly morphin. This acts probably as a primary stimulant directly upon the nerve centers and also as a secondary stimulant by blocking the transmission of shock-producing peripheral stimuli. As a primary stimulant the dose must be small—say one-tenth grain, which may be repeated if necessary. A patient in shock rarely complains of pain. If pain is present, however, or if the shock is perhaps caused by pain, then a full analgesic dose of morphin must be given. Adrenalin and ergot are valuable drugs for the raising of blood pressure, the adrenalin acting particularly well, but its action is evanescent; so the best way to administer the remedy is by repeated small doses, say 10 to 15 minutes of the 1:1000 solution every half hour.

To paraphrase Punch's well-known advice concerning, the best thing to do for shock is to avoid its occurrence. Prevention is far more in the power of the surgeon than is the remedy. In the first place by reassurance and the manifestation of perfect confidence on the part of the surgeon the patient's fears should be quieted. Panic is a well-known contributing cause to shock and is occasionally the sole cause. Even in the case of a patient who shows outward coolness these precautions should not be neglected, for, often through pride, the real state of mind is concealed. The anesthetic, local or general, should be carefully chosen, bearing in mind the fact that consciousness of the steps of an operation may contribute to the occurrence of the shock. Individuals having complicating diseases such as nephritis, diabetes, cardiac disease, etc., should be prepared in a suitable manner, and if there is time such preparations should be thorough. Thus before operations on the urinary system cardiac stimulation, urinary antisepsis and diuresis should be striven for. In particularly nervous persons, even if they have sound hearts, strophanthus or digitalis should be administered for the 24 hours before operation.

In abdominal operations, particularly if large tumors are to be removed, a dose of the salicylate of eserin, 1/40 to 1/32 grain, should be given hypodermically with a little morphin before the patient leaves the table. The eserin is a powerful contractor of circular muscle and acts as an excellent prophylactic against the paralytic distension o fabdominal shock.

Incisions should be as short as is consistent with good surgery—no shorter. No time should be wasted. The time consumed by the opertion should always be noted, though speed, not haste, is to be desired.

These remarks would be incomplete if we failed to call attention to the great value of the two-stage or even the three-stage operation. There is probably no single factor in modern surgery which in suitable cases so surely prevents dangerous shock. It is quite impossible to discuss here the various applications of this principle, but it is sufficient to say that they are more numerous than would at first sight be supposed. To mention one important application we may refer to the two-stage transvesical removal of the prostate, a procedure which diminishes the risk fully 50 per cent.

The prophylaxis of shock is a matter of intelligent experience. Here the skill and judgment of the ripe surgeon make themselves felt. One should know when to stop as well as when and how to begin. Thus the man of judgment will far excel his colleague who possesses mere technical skill. The quick and sure reasoning which looks like intuition will tell the true surgeon when it is wise to refuse to operate, while the mere craftsman, clever though he be, will perform many an incomplete or even worse than useless piece of work.—Howard Lilienthal, in International Journal of Surgery, February, 1907.

